# A Novel Splice Site Variant in the *LDLRAP1* Gene Causes Familial Hypercholesterolemia

**DOI:** 10.52547/ibj.25.5.374

**Published:** 2021-05-15

**Authors:** Najmeh Ahangari, Amirhossein Sahebkar, Mohsen Azimi-Nezhad, Hamideh Ghazizadeh, Mohsen Moohebati, Mahmoud Ebrahimi, Habibollah Esmaeili, Gordon A. Ferns, Alireza Pasdar, Majid Ghayour Mobarhan

**Affiliations:** 1 Department of Medical Genetics and Molecular Medicine, Faculty of Medicine, Mashhad University of Medical Sciences, Mashhad, Iran;; 2 Biotechnology Research Center, Pharmaceutical Technology Institute, Mashhad University of Medical Sciences, Mashhad, Iran;; 3 Neurogenic Inflammation Research Center, Mashhad University of Medical Sciences, Mashhad, Iran;; 4 UMR INSERM U 1122, IGE-PCV “Interactions Gène-Environnement en Physiopathologie CardioVasculaire”, Université de Lorraine, 54000, Nancy, France. School of Medicine, Neyshabur University of Medical Sciences, Neyshabur, Iran;; 5 Student research committee, Mashhad University of Medical Sciences, Mashhad, Iran;; 6 International UNESCO Center for Health-Related Basic Sciences and Human Nutrition, Department of Nutrition, School of Medicine, Mashhad University of Medical Sciences, Mashhad, Iran;; 7 Metabolic Syndrome Research Center, Mashhad University of Medical Sciences, Mashhad, Iran;; 8 Cardiovascular Research Center, School of Medicine, Mashhad University of Medical Sciences, Mashhad, Iran;; 9 Department of Biostatistics & Epidemiology, School of Health, Management & Social Determinants of Health Research Center, Mashhad University of Medical Sciences, Mashhad, Iran;; 10 Division of Medical Education, Brighton and Sussex Medical School, Falmer, Brighton, Sussex, BN1 9PH, UK;; 11 Division of Applied Medicine, Medical School, University of Aberdeen, Forester hill, Aberdeen, UK

**Keywords:** Genetic research, LDLRAP1, Hypercholesterolemia, Hydroxymethylglutaryl-CoA Reductase Inhibitors

## Abstract

**Background:**

FH, a hereditary disorder, is caused by pathogenic variants in the *LDLR*, *APOB*, and *PCSK9* genes. This study has assessed genetic variants in a family, clinically diagnosed with FH.

**Methods:**

A family was recruited from MASHAD study in Iran with possible FH based on the Simon Broom criteria. The DNA sample of an affected individual (proband) was analyzed using WES, followed by bioinformatics and segregation analyses.

**Results:**

A novel splice site variant (c.345-2A>G) was detected in the *LDLRAP1* gene, which was segregated in all affected family members. Moreover, HMGCR rs3846662 g.23092A>G was found to be homozygous (G/G) in the proband, probably leading to reduced response to simvastatin and pravastatin.

**Conclusion:**

*LDLRAP1* c.345-2A>G could alter the PTB, which acts as an important part of biological pathways related to lipid metabolism.

## INTRODUCTION

Familial hypercholesterolemia is an inherited condition due to the pathogenic variants of *LDLR*, *APOB*, and *PCSK9* genes^[^^1^^]^. The most significant complication ‍for FH patient is the high risk of premature coronary heart disease^[^^2^^]^. Based on estimates, the prevalence rate of FH is 1:200 in Western populations; however, over 80% of FH patients remain undiagnosed^[^^3^^]^. 

The World Health Organization has recommended large-scale screening for the identification of FH patients, who will benefit mostly from early treatment with lipid-lowering drugs. Early diagnosis and treatment of these patients can reduce the risk of cardiovascular disease, aiming at lowering the LDL-C concentration, and contribute to the proper management of other risk factors during the life^[^^4^^]^. Therefore, it is crucial to understand the molecular basis of FH to diagnose the disease and manage therapeutic approaches. About 5% of the cases before the age of 60, who have experienced myocardial infarction, is estimated to be heterozygous FH^[^^5^^]^. 

Recent molecular techniques such as WES aim at finding novel genetic variants, which is mainly important in multiethnic populations^[^^6^^]^. The *LDLRAP1* gene or *ARH* is an adapter protein that facilitates the endocytosis of *LDLR* into hepatocytes. Mutations in this gene has been demonstrated to induce a recessive type of FH^[^^7^^]^. It has also been reported that some heterozygous carriers of pathogenic variants in the *LDLRAP1* gene show high LDL-C levels^[^^8^^]^. We describe a novel homozygous splice site variant c.345-2A>G in the *LDLRAP1* gene, which was identified in an FH family.

## MATERIALS AND METHODS

**Subjects and clinical presentation**

This survey is a family-based pedigree study, as a part of a larger research on the genetic assessment of dyslipidemia, MASHAD cohort study, using WES ^[^^9^^]^. Peripheral blood samples were collected, and the sera were separated. Total serum levels of cholesterol, HDL-C, LDL-C, and triglyceride were measured according to a method described previously^[^^9^^]^.

**DNA extraction and WES**

**Fig. 1:**
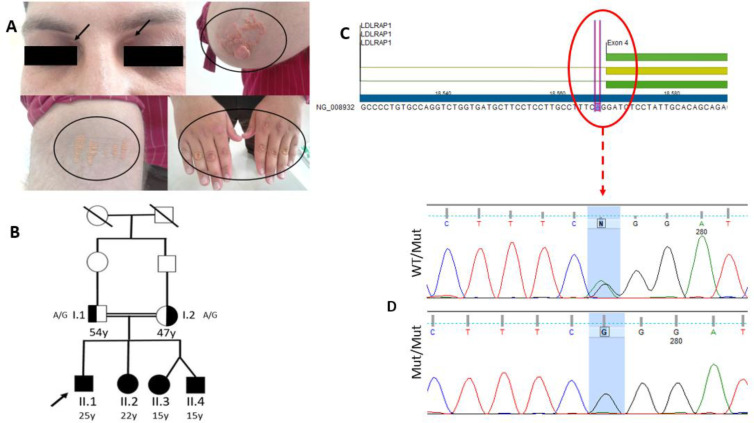
*The clinical feature of FH in the proband. (A) Xanthelasmata formed in the inner canthus of the eyelid as well as xanthomas in elbows and hands (within the circles); (B) pedigree of the family showing a novel *LDLRAP1* variant (c.345-2A>G). The arrow shows the proband; (C) the normal ‘A’ nucleotide at the splice site using CLC workbench v.7.8.1; (D) *LDLRAP1* variant identified in the proband, siblings, and parents. Mut, mutant*

DNA samples were extracted using the standard salting-out method. The quality and quantity of DNA sample were assessed by a Nanodrop (Thermo Scientific, USA) and the genomic DNA extracts were analyzed on the 0.7% agarose gel. WES was performed for the proband’s sample (II.1, Fig. 1) at the Persian Bayan Gene Research and Training Center (Shiraz, Iran). The ES condition was performed as follows: bidirectional sequencing of the complete coding region plus 2-kb upstream and 1-kb downstream, with 150× reads, on an Illumina HiSeq 2500 (Illumina, San Diego, CA, USA). The reads were aligned with the reference genome (hg38) sequences using WinterVar (http://wintervar.wglab.org)^[^^10^^]^. This process was followed by the detection of single nucleotide variants and small insertion and deletions, as well as by the identification of all other variants in the exons of the target genes. Moreover, the effect of nonsynonymous missense variants was predicted using VarSome^[^^11^^]^ and HSF ^[^^12^^]^. Assessment of variants was carried out by computational prediction tools and genetic databases. For further analysis, variants with a minor allele frequency lower than 1% were selected. After the confirmation of the candidate variant, parents and siblings were screened to assess the co-segregation of the suggested variant using PCR and Sanger sequencing. 

**Ethical statement**

The above-mentioned sampling protocol was approved by the Research Ethics Committee of Mashhad University of Medical of Sciences, Mashhad, Iran (ethical code: IR.MUMS.MEDICAL.REC. 1386.250)^[^^9^^]^. All participants have signed the informed consent.

## RESULTS

**Clinical findings**

The proband (II.1, Fig. 1) was a 25-year-old male (with complaints of obesity and skin lesions on his hands and elbows), who was referred to a geneticist. After a detailed physical examination, the proband was found to have a history of two episodes of myocardial infarction at ages 23 and 25 years. Moreover, he had bilateral xanthelasma (Fig. 1). According to Simon Broom Criteria ^[^^13^^]^, he was diagnosed with FH, due to high total cholesterol and LDL-C serum levels before treatment. Other family members consisted of one brother and two sisters who had also high total cholesterol levels and clinical manifestations shown in Table 1. The proband was on treatment with rosuvastatin (40 mg) and ezetimibe (10 mg). Moreover, he took aspirin (80 mg) and bisoprolol (5 mg) for coronary artery disease and hypertension treatment, respectively. The proband responded to the treatment, though he did not reach the lipid target level based on the European Society of Cardiology recommendations. 

**Molecular findings**

Preliminary analysis of the data for the F-10 family revealed that there was a potentially pathogenic variant within the *LDLRAP1* gene: NG_008932.1 (NM_015627.2); c.345-2A>G (GRCh38). This variant caused a nucleotide change at c.345-2A>G, an acceptor splice site in IVS-3 in the *LDLRAP1* gene. The c.345-2A>G was novel as no report was found for this variant in the genomic databases at the time of this study. Moreover, searching for the frequency of this variant in databases such as ExAC (http://exac. broadinstitute.org/), dbSNP (https://www.ncbi.nlm.nih. gov/snp/), and gnomAD (https://gnomad. broadinstitute. org/) yielded no results. To identify the clinical classification of the variant, mutation-related databases, such as ClinVar (https://www.ncbi. nlm.nih.gov/clinvar/), OMIM (https://www. omim.org/), and HGMD (http://www.hgmd.cf.ac.uk/ ac/index.php), were also searched, which no report was found for the c.345-2A>G. The pathogenicity of the c.345-2A>G variant was assessed through HSF, which aims to help the assessment of the pre-mRNA splicing through 12 different algorithms to recognize and predict the effect of nucleotide changes on splicing sites consisting of the acceptor/donor splice sites and the branch point and auxiliary sequences^[^^12^^]^. In the case of a genetic variant, if the WT score is more than the threshold and the score variation (between WT and Mutant) is less than -10% for HSF, it is considered that the breakage of the splice site has occurred. Since c.345-2A>G variation causes -30.94% difference, it is predicted as a breaking site variant. Moreover, this variant was assessed through the VarSome online tool, which allows users to search variants of interest in their genomic context and collects data from multiple databases in a central location, providing free and easy sharing knowledge on human genomic variations^[^^11^^]^. According to all other available tools such as MutationTaster (http://www.mutationtaster.org/), FATHMM-MKL (http://fathmm.biocompute.org.uk/ fathmmMKL.htm), and CADD phred score (https://cadd.gs.washington. edu/), c.345-2A>G was predicted as disease-causing variant. Pathogenicity was also described according to the American College of Medical Genetics and Genomics guidelines^[^^14^^]^. Therefore, c.345-2A>G variant was classified as a ‘pathogenic’ variant. 

**Co-segregation analysis**

The available family members of the proband were segregated for the candidate variant in the *LDLRAP1* gene using Sanger sequencing. The proband (II.1), two sisters (II.2 & II.3) of the proband and one brother (II.4) were homozygous for the *LDLRAP1* gene c.345- 2A>G variants (Fig. 1D). The parents (I.1 & I.2) were heterozygous. The variant segregation results as well as the lipid profile and clinical symptoms are summarized in Table 1.

**Table 1:** *Characteristics of the investigated family carrying novel variant of *LDLRAP1

**Variants & Parameters**	**Father**	**Mother**	**Proband**	**II.2**	**II.3**	**II.3**
*LDLRAP1* variant zygosity	Htz	Htz	Hmz	Hmz	Hmz	Hmz
Age (y)	54	47	25	23	16	16
Gender	M	F	M	F	F	M
Total cholesterol (mg/dL)	177	195	670	610	588	567
Triglyceride (mg/dL)	200	210	126	150	132	161
LDL-C (mg/dL)	135	120	435	389	410	378
HDL-C (mg/dL)	45	51	86	56	59	62
Symptoms	None	None	Xanthelasma, Xanthema, CAD	Xanthelasma, Xanthoma	Xanthelasma, Xanthoma	Xanthelasma, Xanthoma

Hmz, homozygote; Htz, heterozygote; M, male; F, female

**Pharmacogenetics study**

It is well known that statins are the first-line therapy for hypercholesterolemia, though responses to these drugs have shown significant differences among patients. These differences in drug response are partly attributed to the variations in genes involved in pharmacokinetics, pharmacodynamics, and lipid metabolisms^[^^15^^]^ such as *ABCG2*, *SLCO1B1*, *CYP3A4, *and *HMGCR*. In this regard, we analyzed WES data for the best known reported genetic variants associated with lipid-lowering therapeutic response. We found *rs3846662* g.23092A>G, an intronic variant, as homozygous (G/G) in affected individuals. 

## DISCUSSION

In this clinically ascertained patient with FH who had severe hypercholesterolemia, we found an acceptor splice-site mutation (c.345-2A>G) in intron 3 of the* LDLRAP1* gene. This variant was neither reported in any other hypercholesterolemic patients in MASHAD cohort study^[^^16^^,^^17^^]^ nor in the Iranome database (http://www.iranome.ir/). This variant was also absent in ExAC and 1000G databases. The *rs781769339* represented as an A>T substitution has previously been reported as a splice acceptor variant. According to five applied algorithms, this variant was interpreted to alter splicing. The *LDLRAP1* gene encodes a protein consisting of 308 amino acids that involve a PTB (170 amino acids). There are significantly similar sequences to the PTB domains in several adapter proteins. PTB domains, situated in the cytoplasmic domains of several cell surface receptors such as *LDLR*, bind to the NPXY consensus sequence. Exon 4 of *LDLRAP1* is located in the PTB/PID interaction domain^[^^7^^]^. Northern blot analysis has revealed that *LDLRAP1* expression is typically occurred at high levels in the kidney, liver, and placenta, while it is expressed at lower levels in the brain, heart, muscle, colon, spleen, intestine, lung, and leukocytes^[^^18^^]^.

Hypercholesterolemia may be occurred due to failure in the hepatic uptake of LDL in the patient. In a report, the genetic analysis of a Mexican FH family with two affected siblings indicated a new mutation (IVS4 + 2T> G) that affects the donor splice site in* LDLRAP1* IVS-4, while parents and other siblings were heterozygous. Substitution of IVS4 + 2T> G caused another alternative transcript with 78 deleted nucleotides in mature mRNA in the template. Translation of this mRNA led to the production of ARH-26-a mutated protein without 26 amino acids and also the lack of the b6 and b7 strands of the PTB domain. This was the first report of a mutation leading to an altered PTB domain ^[^^19^^]^. Furthermore, increasing LDL uptake by lymphocytes has been reported in individuals carrying *LDLRAP1* mutant ^[^^20^^]^. As described above, we found g.23092A>G (*rs3846662*, an intronic variant) within the *HMGCR* gene in the affected individuals, as well. This variant led to a probably reduced response to simvastatin and pravastatin^[^^21^^]^. Exon 13 of pre-mRNA alternative splicing of *HMGCR* results in two transcripts, known as rs3846662 with full-length *HMGCR* and Δ13 *HMGCR*^[^^22^^]^. *HMGCR* exon 13 encodes a part of the catalytic/statin-binding domain^[^^23^^]^. The *rs3846662* modifies the binding motif of heterozygous nuclear ribonucleoprotein A1, which regulates the alternative splicing of *HMGCR*^[^^24^^]^. It has been suggested that the high amount of Δ13 *HMGCR* mRNA in carriers of the *rs3846662* A allele leads to probable lower activity in *HMGCR*, as well as lower levels of baseline LDL-C and reduced sensitivity and response to the statin inhibition^[^^25^^]^. 

Overall, we describe, herein, an Iranian FH pedigree with a novel splice site acceptor variant in the *LDLRAP1* gene. This variant results in a breaking site in IVS-3 within the PTB domain, whichmay affect the LDLR and also other related receptors. Functional studies and validation of this variant may lead to a more comprehensive FH screening in the future.
